# Metabolites of the Arachidonic Acid Lipoxygenase Pathway May Be Targets for Intervention and Diagnostic Markers for Metabolic Disorders in Pregnancy—A Pilot Study

**DOI:** 10.3390/nu17193170

**Published:** 2025-10-08

**Authors:** Małgorzata Szczuko, Justyna Maj, Kamila Pokorska-Niewiada, Edyta Zagrodnik, Maciej Ziętek

**Affiliations:** 1 Department of Bromatology and Nutritional Diagnostics, Pomeranian Medical University in Szczecin, 70-111 Szczecin, Poland; 60108@student.pum.edu.pl; 2 Department of Toxicology, Dairy Technology and Food Storage, West Pomeranian University of Technology in Szczecin, 71-459 Szczecin, Poland; kamila.pokorska@zut.edu.pl; 3 Clinical Department of Anesthesiology and Intensive Care of Adults and Children, Pomeranian Medical University in Szczecin, 72-010 Police, Poland; edyta.zagrodnik@pum.edu.pl; 4 Department of Perinatology, Obstetrics and Gynecology, Pomeranian Medical University in Szczecin, 72-010 Police, Poland; maciej.zietek@pum.edu.pl

**Keywords:** metabolic disorders, pathology of pregnancy, gestational diabetes, eicosanoids, HETE acids, HODE acids, inflammation mediators

## Abstract

**Background**: Pathological pregnancy is associated with various complications that may affect the health of both the mother and her offspring. In recent years, lipid metabolites such as hydroxyeicosatetraenoic (HETE) fatty acids and hydroxyoctadecadienoic (HODE) fatty acids have gained increasing interest as potential biomarkers of pathological processes in pregnancy. The aims of the present study were to investigate changes in HETE and HODE levels during pathological pregnancy and to assess their potential role in the development and monitoring of pregnancy complications. Attempts were made to determine associations in cross-sectional studies and relationships in longitudinal ones. **Methods**: In this study, a liquid chromatograph (HPLC) was used to separate the eicosanoids. The study group consisted of 72 Caucasian women, divided into a control group (*n* = 51) and a group with non-physiological pregnancy (*n* = 21). **Results**: The study results show that the levels of the tested metabolites of the cyclooxygenase (COX) and lipoxygenase (LOX) pathways increased as pregnancy progressed. Women with non-physiological courses of pregnancy who developed gestational diabetes and/or preeclampsia were characterized by dysregulation of the inflammatory signaling processes involving eicosanoids. **Conclusions**: Carbohydrate abnormalities during pregnancy were mainly associated with increased synthesis of 5-oxoETE and the use of 5-HETE in the control group but were not visible in the diabetic group. HODE acids probably do not play a significant role in pathological pregnancy. The relatively small size of the pathological group and the wide range of gestational age mean that the tests should be standardized and carried out on a larger scale.

## 1. Introduction

Pregnancy induces many adaptive changes in the body’s systems and organs to ensure optimal conditions for fetal development [1]. Dysregulation of these mechanisms causes a change in the concentrations of specific compounds in the body, including fatty acids derived from omega-3 and omega-6. Hydroxyeicosatetraenoic (HETE) fatty acids are formed in the pathway of arachidonic acid metabolism by lipoxygenases, while hydroxyoctadecanoic (HODE) fatty acids are metabolized from linoleic acid [2]. Studies have indicated that the levels of HETE and HODE acids change during pregnancy. Findings are inconclusive and are often limited by the number of patients and variability in the measurement methods. Establishing unambiguous relationships is difficult because their levels are influenced by many factors. With further research, it will be possible to better understand the mechanisms and provide a diagnostic basis for high-risk pregnancies [3]. Precisely determining the date of delivery is not possible due to the inability to precisely determine the moment of conception [4]. We can divide pregnancy into three periods, each of which lasts twelve weeks [5]. During pregnancy, there are dynamic changes in the body, including hormonal, metabolic, respiratory, and cardiovascular changes. A woman’s mood and psyche are also affected during this period [6]. As the fetus grows, the intensity and rate of metabolism and the need for nutrients, vitamins, and minerals increase. Normal weight gain for a pregnant woman is approximately 12 kg, which includes the weight of the baby, the placenta, amniotic and cellular fluids, enlarged mammary glands, and an enlarged uterus, and additional adipose tissue can also be distinguished. A rational diet and the elimination of stimulants in a pregnant woman are important factors in guaranteeing the health of the baby, as well as keeping the mother in good condition [2].

### 1.1. Pregnancy Course and Pathology of Pregnancy

It is known that the course of pregnancy and embryo implantation show characteristic signs of inflammation. These events are associated with the expression of inflammatory mediators, which include cytokines, growth factors, and lipid mediators [7]. During pregnancy, undesirable pathological conditions may occur due to the increased activation of inflammatory pathways or their dysregulation [8]. Pathological changes may include but are not limited to hypertension, preeclampsia (PE), miscarriages, diabetes, intrauterine fetal hypotrophy, and prematurity [9]. Pregnant women diagnosed with hypertension are at increased risk of complications such as early miscarriage, placental abruption, organ failure, and cerebrovascular incidents [10]. Significantly increased blood pressure in a pregnant woman poses a threat to the health and life of the mother, as well as the fetus [11,12]. Developing pregnancy-induced or chronic hypertension with coexisting proteinuria is called preeclampsia. In its mechanism, based on endothelial dysfunction, impaired organ perfusion is observed. It is a multisystem disease characterized by elevated blood pressure with coexisting proteinuria (≥0.3 g/d), thrombocytopenia (defined as a platelet count of less than 100 K/mm), impaired liver function (with liver enzymes greater than two times the upper limit of normal levels), or renal insufficiency (defined as a serum concentration of 1.1 mg/dL or greater). Women with preeclampsia often give birth to premature offspring with fetal growth restrictions [11,13]. Underlying the development of diabetes are changes in carbohydrate metabolism which disrupt adaptive processes, with increasing abnormal insulin resistance (IR) during pregnancy. Even small fluctuations in glycemia, with a tendency towards hyperglycemia, contribute to the development of diabetes [14]. Common abnormalities in newborns from pregnancies complicated by diabetes are defects in the central nervous system and the heart. In the later course of pregnancy, hyperglycemia causes metabolic pathologies that occur after birth, such as hypomagnesemia, hypocalcemia, hypoglycemia, and hyperbilirubinemia [15]. It appears that in both preeclampsia and especially gestational diabetes, modifiable factors are important.

### 1.2. Polyunsaturated Fatty Acids

Fatty acids are a comprehensive group of triacylglycerols. These include the omega-3 family with the main α-linolenic acid (ALA) synthesis pathway and the omega-6 family with the linoleic acid (LA) synthesis pathway [16]. ALA and LA are called essential fatty acids (EFAs) because the human body cannot synthesize them, and they must be supplied externally with food [17]. The right balance between omega-3 and omega-6 fatty acids is important for health. The predominance of omega-6 fatty acids increases the synthesis of arachidonic acid (AA) while inhibiting the synthesis of DHA and EPA. An incorrect ratio of *n*-3 and *n*-6 fatty acids in the diet leads to an imbalance in the physiological balance of the body. Excessive amounts of omega-6 fatty acids relative to omega-3 can contribute to inflammation and increase the risk of heart disease, metabolic disorders, and other health problems [18]. The metabolites of ALA and LA acids are involved in the regulation of body homeostasis [19]. AA is the main precursor of eicosanoids in macrophages and the most common fatty acid in cell membranes [8]. Two enzymes are involved in their formation, cyclooxygenases (COXs), which influence the levels of prostaglandins, prostacyclin, thromboxane, and lipoxygenase (LOX), which is involved in the synthesis of leukotrienes and lipoxins in the HETE acid pathway [20]. On the other hand, HODE acids are also formed from linolenic acid through LOX. The pro-inflammatory role is attributed to 9-HODE, while 13-HODE is a PPARγ agonist and also plays an anti-inflammatory role [21]. In addition, derivatives of the 15 LOX pathway contribute to the regulation of apoptosis [22]. Further, these derivatives may have a muscle relaxation effect, which significantly affects physiological processes, including uterine contractions during labor [23]. Studies have shown that 12-HETE leads to the hyperpolarization of smooth muscle cells [24], and together with other HETE acids, they contribute to the pathogenesis of ischemic heart disease, myocardial hypertrophy, myocardial failure, and the occurrence of atherosclerosis. Recently, it has been shown that AA-derived eicosanoids via cytochrome (CYP) and LOX biosynthesis pathways were positively associated with SGA [25].

### 1.3. The Aim of This Study and Hypothesis

Despite growing interest, the available literature presents contradictory findings regarding the levels of LOX-derived metabolites in pregnancy. Some reports associate elevated concentrations of HETE and HODE with gestational diabetes or preeclampsia, whereas others have failed to demonstrate clear correlations. Major limitations of existing studies are their small sample size, heterogeneity of methodology, and variability in patient populations. Consequently, it is still uncertain whether these lipid mediators could serve as reproducible diagnostic markers or therapeutic targets for pregnancy-related metabolic disorders. The aim of the present pilot study was to evaluate the concentrations of HETE and HODE acids in women with normal pregnancies and those with pregnancy complications, including gestational diabetes. We hypothesized that alterations in the LOX pathway metabolites could reflect metabolic dysregulation in pregnancy and might serve both as early diagnostic biomarkers and as potential targets for intervention in high-risk pregnancies.

## 2. Materials and Methods

Our maternity hospital is one of four in Szczecin and the surrounding area. Patients were recruited over a six-month period (2019) at the Clinic of Perinatology, Obstetrics, and Gynecology SPSK1 Police. Patients who expressed interest in participating in the project represented nearly 20% of women giving birth during that period. Assuming a population of nearly 3500 births per year (for a city with a population of over 400,000), the study sample should have been 76. Due to limited funding, the pilot study was terminated at this stage. Once funding is secured, it will be scaled up. Attempts were made to determine associations in cross-sectional studies and relationships in longitudinal ones.

### 2.1. Characteristics of the Study Group

A total of 72 Caucasian women (100%) were included in the study, and they were between 6 and 37 weeks of gestation, with ages ranging from 21 to 45 years (32.21 ± 5.49). The criterion for including patients in the study was confirmation of pregnancy with a positive pregnancy test (platelet pregnancy tests were used) and ultrasound imaging (GE Voluson E8 BT19, 2016). All pregnancies were singleton and delivered at term (≥38 weeks of gestation). Patients underwent anthropometric examinations, which included measuring their heights and weights. The height ranged from 1.57 m to 1.84 m, with a mean of 1.68 ± 0.056 m. Pre-pregnancy body weight ranged from 49.5 kg to 130.2 kg, and the mean body weight was 84.06 ± 20.79 kg. BMI values were calculated and ranged from 18.43 to 47.67 kg/m^2^, with a mean of 29.75 ± kg/m^2^. The presence of diabetes mellitus and/or pre-diabetes was an additional criterion for the pathological group. The data are shown in Table A1—Appendix A.

The study group was divided into a control group and a non-physiological group. The control group included 51 women, and the non-physiological group included 21 pregnant women. The pregnant patients presented with diabetes mellitus (*n* = 18) and/or preeclampsia (*n* = 3). The comparative characteristics of the two groups are shown in Table 1. The unequal division of groups resulted from the proportion of normal and non-physiological pregnancies during the study period.

### 2.2. Dietary Profile of the Patients

Qualified dietitians collected data to assess dietary intake using Diet v.6.0 software, which is recommended by the Institute of Food and Nutrition in Poland (IZŻ, Warsaw, Poland). There were no significant differences in nutrient intake between groups (Table 2).

### 2.3. Biochemical Parameters

In the study group, basic biochemical tests were performed in a diagnostic laboratory in the following scope (Table 3).

### 2.4. Biochemical Analysis of Eicosanoids

The following components were extracted from plasma samples using RP-18 solid phase SPE extraction columns: 5-HETE, 5-oxoETE, 12-HETE, 15-HETE, HETE 16R/16S, 9-HODE, 13-HODE, 18-HEPE, 17-HDHA, DiDHA 10S/17R, leukotriene B4, prostaglandin E2, prostaglandin B2, lipoxin A4 5S/6R, and lipoxin A4 5S/6R/15R. The extraction of eicosanoids began with precipitation of the proteins from plasma samples by adding 0.5 mL of plasma to 1 mL of acetonitrile and 50 μg of internal standard. The tubes were incubated at negative temperature and then centrifuged (5804R centrifuge). The supernatants were transferred to new tubes and brought to an acidic pH by adding 4.5 mL of 1 mM HCL. In all tubes, the pH was standardized to 3. Activation of the columns was completed by successive washes with 3 mL of 100% acetonitrile and 3 mL of 20% acetonitrile in water. After elution of the eicosanoids, the samples were analyzed by HPLC [26].

Separation was carried out using an Agilent Technologies 1260 liquid chromatograph, consisting of a degasser, column oven, bin pump, and diode array detector. Agilent ChemStation software (Agilent Technologies, Cheadle, UK) was used for instrument control, data acquisition, and analysis. The study was completed using a Thermo Scientific Hypersil BDS C18 column (Agilent Technologies, Cheadle, UK). The column oven was set to 20 °C. A gradient method was used where the mobile phase was a mixture of solvents A and B (methanol/water/acetic acid, 50/50/0.1, *v*/*v*/*v*, and methanol/water/acetic acid, 100/0/0.1, *v*/*v*/*v*, respectively). The amount of buffer B in the mobile phase oscillated between 30% and 98%. The peaks were monitored with a DAD by adsorption at 235 nm for 17-HDHA, 16-HEPE, 13-HODE, 9-HODE, 5-HETE, 12-HETE, and 15-HETE; at 280 nm for 5oxoETE; and at 210 nm for 16-HETE. The peak absorption spectra were analyzed to confirm the identification. The quantification was based on peak areas with internal standard calibration. ChemStation (Agilent Technologies, Cheadle, UK) was used for the quantitative analysis. A detailed description of the methods used was published in 2020 [26].

### 2.5. Statistical Analysis

Statistical analysis was performed using MedCalc^®^ Statistical Software version 22.002 (MedCalc Software Ltd., Ostend, Belgium). The Shapiro–Wilk test was used to check the normality of the distribution. In the data set, the values were scattered and had extreme values, which meant that the set did not follow a normal distribution. Therefore, non-parametric tests were used in the calculations, and the results were presented as medians, quartiles, and standard deviations (medians and SDs). The Mann–Whitney test was used to compare biochemical parameters of both groups. To demonstrate significant differences in the comparison of mediators between the two groups, logarithmic transformation of the data was used. For correlation, a parametric test was used, and the calculations were based on the means and standard deviations. The Pearson test was used to calculate correlations in both groups. The significance of a correlation was determined at a *p*-value of <0.05.

## 3. Results

A lipid profile was determined, and it included total cholesterol, HDL and LDL fractions, and TG. The mean cholesterol concentration was at the normal limit of 202.835 ± 46.7708 mg/dL. In contrast, the mean TG was 152.857 ± 66.7235 mg/dL, and the mean LDL concentration was 125.337 ± 40.6152 mg/dL and was slightly elevated. The HDL fraction was within the reference range. When analyzing carbohydrate metabolism parameters, significant differences were observed between the lowest and highest results for glucose concentrations (50.93–127.09 mg/dL) and insulin concentrations (2.9–105.9 mIU/dL).

In contrast, the averages of these parameters remained normal. The results for glycated hemoglobin were within normal limits. Similarly, the mean results for liver parameters (ALT, AST, and GGTP) were within the reference values.

### 3.1. Comparison of the Groups

Each of the studied parameters was characterized by a large discrepancy between the lowest and highest concentrations (Appendix A). For this reason, the study group was divided into two classifications (control and pathological; Table 3). Significant differences were found in the mean levels in relation to the carbohydrate metabolism parameters (glucose and HbA1c). There was also a trend of higher HDL levels in the control group relative to the pathological group (Table 4).

When comparing the levels of the tested mediators (i.e., HETE and HODE acids), non-significantly higher values of most parameters were found in the control group, except for 5-HETE and 5-oxoETE (Table 5). Statistically significant differences between both groups were not demonstrated, but the trend was visible in relation to 5-oxoETE and 5-HETE.

### 3.2. Correlation Results

The correlations between the levels of fatty acid derivatives and anthropometric parameters were numerous and of weak strength. Some of them were statistically significant. Most correlations were observed between the week of gestation and the levels of the tested eicosanoids, in particular, 9 and 13 HODE and 15-HETE. Significant correlations were also observed for body weight and BMI with 5-HETE. The data are presented in Table A2—Appendix A.

In the study group, there were no significant correlations between the derivatives and the biochemical parameters (Table A3—Appendix A).

The correlations between the levels of fatty acid derivatives and the anthropometric and biochemical parameters for the total study group were numerous and of weak strength. Several of them proved to be statistically significant, including the week of gestation with 9 and 13 HODE and 5- and 15-HETE, and BMI and body weight with 5-HETE (Table 6).

Of the biochemical parameters, only the correlation between glycated hemoglobin and 12S HETE proved to be statistically significant. A certain trend was observed between glycated hemoglobin and 5-HETE and between fasting glucose levels and 5-oxoETE. The data are shown in Table 7.

The analysis of the correlations between the studied mediators and the anthropometric parameters in the non-physiological group showed several correlations of weaker strength and three of medium strength. Only the week of gestation in relation to 9 HODE and 15s HETE proved to be statistically significant. A trend against 13 HODE was evident. The results are shown in Table 8.

None of the biochemical parameters showed significant correlations with the lipid mediator derivatives (Table A4—Appendix A). A certain trend was observed between CRP and 5-HETE levels and between TG levels and 5-oxoETE (Table 9).

## 4. Discussion

Scientific investigations have unquestionably confirmed the effects of eicosanoids on the vascular and immune systems, renal function, the occurrence of diabetes or preeclampsia associated with hypertension, and renal dysfunction [27]. There is a growing interest in the lipid derivatives of HETE and HODE, which has led to the possibility of using these acids as potential biomarkers for therapeutic targets. When comparing the levels of the tested mediators (i.e., HETE and HODE acids), non-significantly higher values of most parameters were found in the control group, except for 5-HETE and 5-oxoETE (Table 5). The results obtained so far in studies are difficult to interpret for a number of reasons. The formulation of unambiguous conclusions from the studies is particularly difficult given the diversity of the study materials (e.g., placenta, maternal blood, maternal plasma, umbilical cord blood, and amniotic fluid) and the differently designed methods and periods of collection of the biological materials studied (e.g., perimplantation period; first, second, or third trimester of gestation; perinatal period; and puerperium). Therefore, it appears that the final clarification of some issues will continue be the subject of research analysis and project implementation for a long time to come. HETE and HODE can be thought of as mere byproducts in the formation of more complex eicosanoids such as leukotrienes and prostaglandins. It is clear that at least some of them have their own distinct actions and pathophysiological roles. Among the pathologies of pregnancy, PE has been the most thoroughly studied to date, and the involvement of some eicosanoids in this pregnancy pathology has been indisputably proven [28,29]. It is also known from the published literature that increased placental production of HETE acids occurs in preeclampsia, and they can serve as biomarkers for predicting spontaneous preterm labor [28].

In our study, we omitted the CYP 450 metabolites of the eicosanoid synthesis pathway. However, other authors have reported elevated levels of 20-HETE and SPM pathway intermediates, i.e., 14-HDHA, 18-HEPE, and 17-HDHA, at 32 weeks of gestation in women with diabetes and preeclampsia [30]. The eicosanoid with the strongest association with pregnancy pathology was 12-HETE, a product of the LOX pathway, and 12-HETE is a potent pro-inflammatory chemoattractant for neutrophils that causes cytoskeletal reorganization, cytokine production, and endothelial cell adhesion [30]. Therefore, we decided to investigate other mediators of the LOX and COX pathways. Among the LOX enzymes, 15-LOX has been shown to positively regulate cell growth and implantation, and it also contributes to cancer, atherosclerosis, and immune responses [31,32], while 15-HETE acid inhibits the formation of pro-inflammatory leukotrienes and increases granulocyte and lymphocyte activity [33]; further, 15S-HETE may promote protective effects against cancer [34]. The main product of the endometrium, myometrium, and placenta is 12-HETE, while amniotic membranes produce 5-HETE in the highest amount [35]. Higher concentrations of 12- and 15-HETE have been observed in amniotic fluid during labor compared with women who did not start labor [36], and the umbilical cord blood of preeclamptic women contains significantly higher serum concentrations of 15-HETE than healthy pregnant women [37]. In addition, it was found that 12-HETE and other metabolites of the LOX pathway may be involved in the regulation of progesterone biosynthesis in the placenta. Their higher concentrations reduced progesterone levels [38]. The eicosanoids formed from 12/15-LOX have been found to be involved in various inflammatory and physiological pathways such as thrombocyte aggregation, lymphocyte activation and migration, leukocyte chemotactic stimulation, cancer cell metastasis, and cell apoptosis [39]. In turn, higher concentrations of 5-HETE and 15-HETE and lower 9-HETE levels are associated with a higher risk of preterm labor [40]. The total HETE concentrations in the placentas of pregnant women with insulin-dependent diabetes mellitus increase dramatically. Studies have shown that the plasma samples of patients with diabetes are characterized by higher concentrations of 12-HETE, 5-HETE, and 20-HETE [41,42].

### 4.1. Main Findings and Comparison with Previous Studies

In this pilot study, we observed an increase in 5-oxoETE and a decrease in 5-HETE in women with pathological pregnancies. This pattern suggests enhanced conversion of 5-HETE into 5-oxoETE, a more reactive metabolite. We also found that 5-oxoETE was positively correlated with glucose and triglyceride levels, while 12-HETE was associated with HbA1c, indicating a possible link between LOX-derived mediators and metabolic dysregulation in pregnancy. Our findings are consistent with earlier reports that demonstrated higher concentrations of LOX metabolites, including 12-HETE and 5-HETE, in patients with diabetes or preeclampsia [36,41,42,43]. Other studies have also reported elevated levels of 12- and 15-HETE in amniotic fluid and cord blood in complicated pregnancies [36,37], suggesting that our results are in line with previous observations across different populations, although direct ethnic comparisons were not performed in our study because the inhabitants of Poland are a homogeneous Caucasian group. Evidence on HODE metabolites remains limited, but our data suggest only weak associations with gestational age, which aligns with the notion that their role in pregnancy may be secondary compared with HETE acids.

### 4.2. Clinical Implications

The associations of 5-oxoETE with markers of inflammation and lipid metabolism and of 12-HETE with long-term glycemic status support the hypothesis that these metabolites could be useful as diagnostic biomarkers of metabolic complications in pregnancy. Identifying such markers early could help clinicians stratify risk, particularly for gestational diabetes or preeclampsia, and may open the possibility of targeted interventions aimed at modulating LOX pathways.

Future studies should include larger, multi-ethnic cohorts with standard sampling covering all trimesters of pregnancy. Longitudinal designs are particularly needed to clarify the cause–effect relationships between LOX metabolites and pregnancy complications. Mechanistic studies could also help to determine whether 5-oxoETE and 12-HETE are simply markers of dysregulation or active contributors to disease processes. Such studies are necessary before these mediators can be considered reliable biomarkers or therapeutic targets in clinical practice. In our study, we found that the 9 HODE, 15 HETE, and 13-HODE pathways were associated in the pathological group with the week of gestation (Table 7) without playing a role in the correlations. 5 HETE and 5-oxoETE were correlated with the occurrence of inflammation (CRP) and the level of triglycerides stored by adipocytes in obese women. Therefore, in our opinion, carbohydrate disturbances during pregnancy are mainly associated with increased synthesis of 5-oxoETE and 12-HETE. Our finding that 5-oxoETE and 12-HETE may be targets for intervention and that 5-oxoETE may also be a diagnostic marker of metabolic disorders in pregnancy is based mainly on observational changes, which may constitute a limitation of this study.

### 4.3. Strengths and Limitations

The strength of this study is that it provides novel insights into the role of LOX-derived lipid mediators in pregnancy complications using direct biochemical measurements. However, several limitations must be acknowledged. First, the study was based on a very small sample size, limiting statistical power. Secondly, the cross-sectional design precludes conclusions about causality. Third, the wide range of gestational ages at sampling introduced heterogeneity, reducing the comparability of results. Finally, the findings may not be generalizable to other populations and their external validity remains limited. Other limitations of the study include the relatively small sample size and the wide range of gestational ages. More standardized sampling points would have reduced the variability.

## 5. Conclusions

The study of HETE and HODE levels during pathological pregnancy is important for better understanding the non-physiological mechanisms occurring in mothers’ bodies and their impacts on fetal health. The growing interest in lipid compounds has opened up the possibility of using these acids as potential biomarkers or mediators of pathological processes in pregnancy. Of the mediators we investigated, two (5-oxoETE and 12-HETE) could be targets for intervention, and 5oxo ETE could also be a diagnostic marker for metabolic disorders in pregnancy—further testing is necessary to confirm the results. In addition, we showed that the group of women with higher body weights who developed gestational diabetes and/or preeclampsia was characterized by the dysregulation of inflammatory signaling processes involving eicosanoids, which may be the main reason for the lack of well-balanced pregnancies. HODE acids appear to be less associated with abnormal pregnancy outcomes (diabetes) than HETE acids.

## Figures and Tables

**Table 1:** Anthropometric characteristics of the control and pathological groups.

Parameter	Control Group (CG)				Pathological Group (PG)				*p*-Value
	Mean	SD	Min	Max	Mean	SD	Min	Max	
Age (years)	31.94	5.81	21.04	45.03	32.37	5.01	21.42	41.36	0.777
Height (m)	1.69	0.06	1.57	1.84	1.66	0.05	1.57	1.74	0.042
Body weight (kg)	80.03	19.69	49.00	130	98.1	14.35	68.03	125.6	0.0007
Week of gestation (week)	19.21	8.61	6.00	37.04	20.52	8.74	6.04	36.08	0.570
BMI (kg/m^2^)	27.95	6.59	18.44	43.03	36.02	5.91	25.29	47.86	0.00002

**Table 2:** The comparison of mean nutrient consumption.

Nutrient	PG Avg ± SD	CG Avg ± SD	*p*
Energy (kcal)	2168.62 ± 941.89	2180.81 ± 574.11	0.716
Total proteins (g)	89.16 ± 38.42	91.12 ± 25.63	0.614
Animal protein (g)	60.56 ± 32.56	58.87 ± 19.48	0.798
Plant protein (g)	28.22 ± 11.44	28.93 ± 10.05	0.540
% energy from protein	16.78 ± 3.82	16.53 ± 3.73	0.932
Fat (g)	89.91 ± 46.08	92.38 ± 36.52	0.540
SFAs (g)	38.73 ± 22.06	31.71 ± 11.19	0.189
MUFAs (g)	33.06 ± 20.32	38.04 ± 18.68	0.248
PUFAs (g)	11.27 ± 8.18	15.32 ± 9.42	0.212
EPA (mg)	0.12 ± 0.14	0.19 ± 0.53	0.212
DHA (mg)	0.13 ± 0.23	0.65 ± 1.49	0.045
Cholesterol (mg)	282.51 ± 173.08	316.12 ± 199.39	0.489
% energy from fat	35.57 ± 9.89	36.29 ± 8.34	0.636
Total carbohydrates (g)	265.32 ± 128.52	275.57 ± 114.42	0.407
Assimilable carbohydrates (g)	242.12 ± 121.81	248.63 ± 106.59	0.603
Dietary fiber (g)	23.13 ± 10.78	27.13 ± 11.92	0.160
Saccharose (g)	53.55 ± 62.88	39.52 ± 25.44	0.327
% energy from carbohydrates	45.53 ± 9.83	44.78 ± 8.28	0.582

**Table 3:** Biochemical parameters used and their reference values.

Parameter	Abbreviation	Reference Values
C-reactive protein	CRP	<3.1 mg/L
Fasting glucose	GLU	70–99 mg/dL
Insulin	INS	5–25 mU/L
Glycated hemoglobin	HbA1	<5.7%
Total cholesterol	ChT	<190 mg/dL
Cholesterol fraction, HDL	HDL	≥45 mg/dL
Cholesterol fraction, LDL	LDL	<115 mg/dL
Triglycerides	TG	≤150 mg/dL
Alanine aminotransferase	ALT	0–33 U/L
Aspartate aminotransferase	AST	0–32 U/L
Gamma-glutamyltranspeptidase	GGTP	5–35 U/L

**Table 4:** Biochemical results for each group.

Parameters	Control Group				Pathological Group				*p*-Value
	Mean	SD	Min	Max	Mean	SD	Min	Max	
CRP (mg/L)	6.45	5.16	1.17	20.8	8.73	5.33	2.87	20.85	0.130
GLU (mg/dL)	79.41	9.23	50.92	96.9	85.82	14.82	63.5	127.1	0.038
INS (mU/L)	13.71	11.05	2.87	80.2	28.62	22.80	6.6	105.9	0.509
HbA1c (%)	5.08	0.31	4.51	5.74	5.31	0.29	4.75	5.94	0.041
ChT (mg/dL)	201.21	42.62	128.91	310.4	208.32	56.23	132.6	324.8	0.563
HDL (mg/dL)	70.91	13.78	37.43	103.4	62.98	15.66	46.18	100.9	0.073
LDL (mg/dL)	122.19	36.69	61.83	220.8	137.11	51.23	69.43	258.5	0.227
TG (mg/dL)	145.62	67.94	54.59	413.7	171.88	58.79	98.59	281.8	0.191
GGTP (U/L)	11.71	9.71	4	48	15.71	14.64	4	66	0.217
ALT (U/L)	18.23	15.21	5	85	19.23	14.23	5	58	0.942
AST (U/L)	16.93	8.31	10	51	17.42	6.07	9	34	0.460

**Table 5:** Comparison of the mediator levels (i.e., HETE and HODE acids) in both groups.

Parameter (μg/mL)	Control Group Mean ± SD	Pathological Group Mean ± SD	*p*-Value
13S HODE	0.004 ± 0.004	0.004 ± 0.003	0.673
9S HODE	0.004 ± 0.005	0.003 ± 0.003	0.287
15S HETE	0.063 ± 0.068	0.050 ± 0.039	0.261
12S HETE	0.068 ± 0.065	0.051 ± 0.026	0.111
5-oxoETE	0.025 ± 0.331	0.038 ± 0.044	0.064
5-HETE	0.049 ± 0.060	0.033 ± 0.019	0.095

**Table 6:** Correlations between PUFA derivatives and the anthropometric parameters for the control group.

Eicosanoids (μg/mL)		Age (Years)	Height (m)	Body Mass (kg)	Week of Gestation (Week)	BMI (kg/m^2^)
13S HODE	R	0.128	−0.276	−0.177	0.317	−0.112
	*p*-Value	0.374	0.057	0.233	0.023	0.455
9S HODE	R	0.126	−0.297	−0.226	0.312	−0.158
	*p*-Value	0.382	0.041	0.127	0.026	0.289
15S HETE	R	0.259	−0.263	−0.189	0.293	−0.134
	*p*-Value	0.069	0.071	0.202	0.037	0.368
12S HETE	R	0.286	−0.086	−0.173	0.274	−0.166
	*p*-Value	0.044	0.563	0.244	0.051	0.265
5-oxoETE	R	0.097	−0.205	0.1	0.187	0.18
	*p*-Value	0.505	0.161	0.502	0.189	0.226
5-HETE	R	0.173	−0.199	−0.312	0.28	−0.289
	*p*-Value	0.23	0.175	0.033	0.047	0.049

Abbreviations: R, Pearson correlation coefficient (r).

**Table 7:** Correlations between PUFA derivatives and the biochemical parameters in the control group.

Parameter		13S HODE (μg/mL)	9S HODE (μg/mL)	15S HETE (μg/mL)	12S HETE (μg/mL)	5-oxoETE (μg/mL)	5-HETE (μg/mL)
CRP (mg/L)	R	−0.102	−0.133	−0.148	−0.082	−0.084	−0.075
	*p*-Value	0.486	0.360	0.310	0.574	0.568	0.607
GLU (mg/dL)	R	0.005	−0.069	−0.054	−0.064	**0.248**	−0.143
	*p*-Value	0.972	0.633	0.710	0.660	**0.082**	0.323
INS (mU/L)	R	0.002	0.004	−0.022	−0.081	0.022	0.015
	*p*-Value	0.991	0.978	0.882	0.576	0.882	0.919
HbA1c (%)	R	−0.135	−0.137	−0.148	**−0.296**	0.004	**−0.281**
	*p*-Value	0.372	0.362	0.325	**0.045**	0.981	**0.059**
ChT (mg/dL)	R	0.064	0.076	0.142	0.148	0.031	0.077
	*p*-Value	0.654	0.598	0.321	0.3013	0.827	0.590
HDL (mg/dL)	R	0.015	0.021	0.052	0.058	−0.06	0.064
	*p*-Value	0.915	0.884	0.717	0.685	0.676	0.654
LDL (mg/dL)	R	−0.009	0	0.074	0.09	0.001	−0.004
	*p*-Value	0.952	1.0	0.603	0.529	0.995	0.979
TG (mg/dL)	R	0.174	0.202	0.21	0.19	0.196	0.176
	*p*-Value	0.227	0.221	0.144	0.186	0.174	0.221
GGTP (U/L)	R	0.011	0.095	0.139	0.029	0.068	0.171
	*p*-Value	0.942	0.522	0.346	0.844	0.646	0.246
ALT (U/L)	R	−0.62	−0.019	−0.053	−0.076	−0.087	0.083
	*p*-Value	0.668	0.897	0.711	0.597	0.542	0.564
AST (U/L)	R	0.031	0.068	0.029	0.031	−0.093	0.173
	*p*-Value	0.827	0.637	0.842	0.831	0.516	0.225

Abbreviations: R, Pearson correlation coefficient (r). Boldface highlights statistically significant results.

**Table 8:** Correlations between PUFA derivatives and the anthropometric parameters in the pathological group.

Eicosanoids (μg/mL)		Age (Years)	Height (m)	Body Mass (kg)	Week of Gestation (Week)	BMI (kg/m^2^)
13S HODE	R	0.181	−0.169	0.214	0.438	0.255
	*p*-Value	0.473	0.503	0.409	**0.061**	0.323
9S HODE	R	0.088	−0.17	0.289	0.49	0.337
	*p*-Value	0.729	0.500	0.260	0.033	0.186
15S-HETE	R	0.235	−0.009	0.239	0.464	0.232
	*p*-Value	0.348	0.970	0.355	0.045	0.371
12S HETE	R	−0.027	−0.085	0.323	0.146	0.296
	*p*-Value	0.915	0.737	0.200	0.550	0.248
5-oxoETE	R	0.131	−0.157	0.208	0.338	0.217
	*p*-Value	0.604	0.534	0.423	0.157	0.402
5-HETE	R	−0.165	0.136	−0.097	0.099	−0.107
	*p*-Value	0.512	0.590	0.712	0.688	0.684

Abbreviations: R, Pearson correlation coefficient (r). Boldface highlights statistically significant results.

**Table 9:** Correlations between PUFA derivatives and the biochemical parameters in the pathological group.

Parameter		13S HODE (μg/mL)	9S HODE (μg/mL)	15S HETE (μg/mL)	12S HETE (μg/mL)	5-oxoETE (μg/mL)	5-HETE (μg/mL)
CRP (mg/L)	R	0.018	0.067	−0.06	−0.024	−0.24	**0.467**
	*p*-Value	0.946	0.807	0.824	0.928	0.370	**0.068**
GLU (mg/dL)	R	0.085	0.092	0.308	0.115	0.229	0.244
	*p*-Value	0.747	0.726	0.228	0.660	0.377	0.345
INS (mU/L)	R	−0.085	−0.108	−0.002	−0.103	0.074	−0.16
	*p*-Value	0.746	0.681	0.995	0.693	0.779	0.540
HbA1c (%)	R	−0.264	−0.23	0.054	−0.146	−0.081	0.143
	*p*-Value	0.342	0.409	0.850	0.603	0.775	0.612
ChT (mg/dL)	R	0.055	0.18	0.103	0.059	0.148	−0.171
	*p*-Value	0.833	0.489	0.694	0.823	0.571	0.512
HDL (mg/dL)	R	−0.065	0.065	0.001	−0.159	−0.097	0.215
	*p*-Value	0.804	0.805	0.996	0.543	0.710	0.407
LDL (mg/dL)	R	0.022	0.103	0.019	0.096	0.122	−0.314
	*p*-Value	0.932	0.694	0.941	0.713	0.641	0.220
TG (mg/dL)	R	0.31	0.364	0.349	0.279	**0.472**	−0.156
	*p*-Value	0.226	0.151	0.170	0.278	**0.056**	0.549
GGTP (U/L)	R	−0.145	−0.063	−0.052	−0.184	−0.259	0.125
	*p*-Value	0.578	0.810	0.842	0.480	0.316	0.632
ALT (U/L)	R	−0.146	−0.171	−0.245	−0.3	−0.371	−0.012
	*p*-Value	0.575	0.511	0.343	0.243	0.143	0.962
AST (U/L)	R	−0.207	−0.193	−0.246	−0.217	−0.293	0.039
	*p*-Value	0.425	0.458	0.342	0.2922	0.253	0.881

Boldface highlights statistically significant results.

## Data Availability

Dataset available on request from the authors.

## Appendix A

**Table A1:** Anthropometric characteristics of the study group (total).

Parameter	Mean	SD	Min	Max
Age (years)	31.71	5.99	21	44
Height (m)	1.68	0.06	1.57	1.83
Body weight (kg)	83.16	20.9	49.5	130.2
Week of gestation (week)	19.72	8.52	6.0	36
BMI (kg/m^2^)	29.25	7.17	18.44	47.86

Abbreviations: SD, standard deviation; Min, minimum; Max, maximum.

**Table A2:** Biochemical results of the studied pregnant women (total).

Parameter	Mean	SD	Min	Max
CRP (mg/L)	6.95	5.27	0.8	20.85
Glucose (mg/dL)	82.61	11.03	50.93	127.09
Insulin (mU/L)	18.12	16.52	2.9	105.9
HbA1c (%)	5.21	0.333	4.51	5.94
Total cholesterol (mg/dL)	203.4	47.81	128.9	324.86
Cholesterol fraction HDL (mg/dL)	68.21	14.39	43.07	103.4
Cholesterol fraction LDL (mg/dL)	124.64	41.615	61.8	258.5
Triglycerides (mg/dL)	158.61	62.23	54.59	413.66
GGTP (U/L)	12.82	12.29	4	66
ALT (U/L)	18.65	14.68	5	85
AST (U/L)	17.27	8.08	10	51

CRP—C-reactive protein; GLU—fasting glucose; INS—insulin; HbA1c—glycated hemoglobin; ChT—total cholesterol; HDL—cholesterol fraction, HDL; LDL—cholesterol fraction, LDL; TG—triglycerides; GGTP—gamma-glutamyltranspeptidase; ALT—alanine aminotransferase; ASP—aspartate aminotransferase.

**Table A3:** Correlations between derivatives and anthropometric parameters for the total study group.

Eicosanoid (μg/mL)		Age (Years)	Height (m)	Body Mass (kg)	Week of Gestation (Week)	BMI (kg/m^2^)
13S HODE	R	0.135	−0.242	−0.126	0.328	−0.06
	*p*-Value	0.273	0.050	0.322	0.005	0.639
9S HODE	R	0.118	−0.245	−0.182	0.325	−0.115
	*p*-Value	0.336	0.047	0.150	0.006	0.364
15S HETE	R	0.252	−0.189	−0.162	0.307	−0.116
	*p*-Value	0.038	0.128	0.201	0.010	0.360
12S HETE	R	0.24	−0.049	−0.162	0.232	−0.154
	*p*-Value	0.048	0.695	0.202	0.053	0.224
5-oxoETE	R	0.104	−0.218	0.18	0.243	0.245
	*p*-Value	0.400	0.078	0.155	0.043	0.051
5-HETE	R	0.135	−0.122	−0.319	0.23	−0.294
	*p*-Value	0.272	0.328	0.010	0.056	0.018

**Table A4:** Correlations between fatty acid derivatives and the biochemical parameters for the total study group.

Parameter		13S HODE (μg/mL)	9S HODE (μg/mL)	15S HETE (μg/mL)	12S HETE (μg/mL)	5-oxoETE (μg/mL)	5-HETE (μg/mL)
CRP (mg/L)	R	−0.091	−0.115	−0.146	−0.094	−0.097	−0.046
	*p*-Value	0.473	0.360	0.2445	0.457	0.441	0.716
GLU (mg/dL)	R	0.004	−0.054	0.006	−0.059	0.265	−0.102
	*p*-Value	0.973	0.666	0.960	0.637	0.030	0.413
ALT (U/L)	R	−0.077	−0.045	−0.086	−0.103	−0.162	0.064
	*p*-Value	0.535	0.713	0.483	0.402	0.186	0.605
AST (U/L)	R	0.005	0.041	−0.001	0.009	−0.14	0.165
	*p*-Value	0.965	0.737	0.996	0.939	0.254	0.1795
ChT (mg/dL)	R	0.056	0.086	0.123	0.117	0.08	0.037
	*p*-Value	0.647	0.488	0.318	0.343	0.515	0.766
HDL (mg/dL)	R	0.013	0.048	0.059	0.051	−0.101	0.098
	*p*-Value	0.916	0.699	0.633	0.682	0.414	0.426
LDL (mg/dL)	R	−0.011	0.006	0.047	0.066	0.061	−0.049
	*p*-Value	0.932	0.962	0.702	0.593	0.622	0.692
TG (mg/dL)	R	0.182	0.206	0.212	0.173	0.271−	0.138
	*p*-Value	0.141	0.095	0.084	0.161	0.026	0.264
GGTP (U/L)	R	−0.039	−0.115	0.059	−0.038	−0.10	−0.046
	*p*-Value	0.755	0.360	0.643	0.765	0.441	0.716
INS (mU/L)	R	−0.044	−0.063	−0.05	−0.116	0.101	−0.066
	*p*-Value	0.721	0.615	0.686	0.352	0.415	0.594
HbA1c (%)	R	−0.154	−0.173	−0.113	−0.298	0.035	−0.256
	*p*-Value	0.236	0.184	0.384	0.020	0.791	0.047

Abbreviations: R, Pearson correlation coefficient (r).
